# Activity patterns of primary school children during participation in The Daily Mile

**DOI:** 10.1038/s41598-021-86631-2

**Published:** 2021-04-02

**Authors:** Lorna M. Hatch, Ryan A. Williams, Karah J. Dring, Caroline Sunderland, Mary E. Nevill, Simon B. Cooper

**Affiliations:** Department of Sport Science, Exercise and Health Research Group, Sport, Health and Performance Enhancement (SHAPE) Research Centre, Nottingham Trent University, Nottingham, UK

**Keywords:** Physiology, Anatomy

## Abstract

The Daily Mile is a popular physical activity initiative in primary schools, yet little is known regarding the activity patterns of children during The Daily Mile. Eighty children (10.4 ± 0.7 years) participated in The Daily Mile (20-min). Activity patterns were assessed using global positioning systems (total distance and age-specific speed zones), alongside heart rate. Cardiorespiratory fitness was assessed using the Multi-Stage Fitness Test. Participants covered a distance of 2511 ± 550 m during The Daily Mile, and heart rate was 163 ± 27 beats^.^min^−1^. Participants travelled the furthest distance, and were most intermittent, during the first 5 min (main effect of time, both *p* < 0.001). Boys ran further and their activity was more intermittent compared to girls (main effect of sex, both *p* < 0.001). Moreover, the highest fit children ran further than less fit children (main effect of fitness, *p* < 0.001). This study provides novel evidence of the nature of physical activity during The Daily Mile; demonstrating that children covered, on average, 1.5-miles and exercised at a moderate-to-vigorous intensity. Furthermore, boys covered a greater distance and were more intermittent than girls; whilst higher fit children ran further than lower fit children. In summary, The Daily Mile makes a valuable contribution to in-school physical activity targets in all children.

## Introduction

Since its development in 2012, the school-based physical activity (PA) initiative The Daily Mile^TM^, has gained research interest as it has been widely and rapidly adopted and has received substantial funding. Currently, The Daily Mile is implemented in more than 11,900 schools in over 79 countries^1^. Moreover, £1.5 million of National Lottery money has been provided to implement the initiative in every primary school in the United Kingdom^2^. The initiative involves children completing ~ 1 mile (approximately 15–20 min) of self-paced PA (i.e. walking, jogging, running, sprinting) each day; typically comprising of laps of an outdoor playground or sports pitch. In recent years, the effect of both acute and chronic participation in The Daily Mile on young people has been examined. Emerging evidence suggests that chronic participation in The Daily Mile over a period of six months results in beneficial effects for both adiposity (1.4 mm reduction in sum of skinfolds) and physical fitness (40 m increase in multi-stage fitness test performance), when compared to children who did not participate in The Daily Mile^3^. With regards to cognitive function, acute participation in The Daily Mile has been suggested to enhance subsequent inhibitory control and verbal working memory^4^; though ambiguity remains as others reported no effect on attention, shifting, inhibitory control or working memory^5^.

To better understand the impact of The Daily Mile on health and cognitive outcomes in young people, quantification of the PA undertaken is required. The need for quantification of the PA undertaken is supported by previous research which shows that the intensity and modality of PA, for example, affect the acute and chronic effects of PA on both health and cognition^6–9^. Moreover, determining the activity patterns of children during participation in The Daily Mile will also establish how the activity contributes to government PA guidelines^10^. Given the multitude of PA interventions available to schools, it is important to fully understand the ‘dose’ of PA involved and subsequent effects on health and cognition, to ensure that the most holistically beneficial intervention is implemented.

Although qualitative studies have sought to explore the dose of PA accrued during school-based running programmes such as The Daily Mile^11,12^, only one study to date has quantitatively examined children’s PA during The Daily Mile^5^. Specifically, the study used accelerometery to assess time spent in moderate to vigorous physical activity (MVPA)^5^. The authors noted that children (9.0 ± 0.5 years) engaged in 10.7 ± 2.7 min of MVPA during participation in The Daily Mile. However, large variability between individuals was found. For example, the most active child spent the entire duration (15 min) of The Daily Mile engaged in MVPA (achieving 50% of the Department of Health and Social Care target of 30 min in-school MVPA per day), yet the least active child spent only 33% of The Daily Mile engaged in MVPA (accumulating only 17% of the 30 min in-school target)^13^. While valuable, these findings highlight the need for additional research which provides a more comprehensive explanation of the activity patterns of children during The Daily Mile.

It is also important to consider how inter-individual differences, such as sex and physical fitness, may influence the PA patterns of young people during The Daily Mile. With regards to sex, it is well established that boys are more physically active than girls during both childhood^14^ and adolescence^15^. Moreover, a number of studies have examined the activity patterns of children during school-based PA^16–19^. One study, for example, reported that girls engaged in more sedentary time (40% vs. 30%) and light PA (36% vs. 33%) during recess, whilst boys engaged in more moderate (20% vs. 27%) and vigorous (4% vs. 11%) PA^20^. Moreover, boys have been reported to spend more time in MVPA during physical education classes, compared to girls (42% vs. 35%)^21^. However, contrasting evidence demonstrated that girls participated in more MVPA during recess periods compared to boys (38% vs. 31% of recess time, respectively)^22^. Taken together, these findings suggest that it is vital that sex is considered in research which aims to investigate and/or evaluate PA interventions in young people. Moreover, a recent study found that boys travelled a greater distance compared to girls during an extracurricular running-based intervention, Marathon Kids, where children aimed to run a Marathon over an academic year^19^. To date however, no research has examined how participant sex may influence the activity patterns of children during participation in The Daily Mile. Moreover, no studies have explored whether other aspects of children’s activity patterns (e.g. speed and the intermittent vs. continuous nature of PA) may be different between the sexes during participation in PA interventions.

Furthermore, it is well documented that repeated participation in PA enhances cardiorespiratory fitness in young people^23^. A cross-sectional study, for example, confirmed an association between PA (measured using accelerometery) and physical fitness (measured via an indirect maximal cycle ergometer test) in 9 and 15 year old children^24^. Moreover, a six-month school-based intervention study reported an improvement in aerobic fitness (measured using the 20 m shuttle run test) in children (6–10 years) who participated in two additional 60 min moderate intensity PA sessions a week (baseline: level 3 ± 1; follow up: level 4 ± 1), compared to controls (baseline: level 3 ± 1, follow up: level 3 ± 1)^25^. However, to our knowledge, there is no empirical evidence examining how physical fitness affects free-living PA and no study to date has examined the effect of physical fitness on PA patterns during The Daily Mile. Knowledge of the impact of physical fitness on activity patterns during The Daily Mile would facilitate further understanding of the absolute and relative ‘dose’ of PA received by each individual and the likely subsequent effects on important outcomes such as health and cognitive performance.

Therefore, the aim of the present study is to examine the activity patterns of primary school children during The Daily Mile and to explore the potential moderating role of participant sex and cardiorespiratory fitness on the activity patterns of children during participation.

## Methods

### Design

This cross-sectional descriptive study involved a familiarisation trial, followed by a main trial. At the beginning of the familiarisation trial (~ 9.00 am), anthropometric measures of height, body mass, waist circumference, skinfolds and sitting height were taken. A Leicester Height Measure (Seca, Hamburg, Germany), accurate to 0.1 cm, was used to measure height and a Seca 770 digital scale (Seca, Hamburg, Germany), accurate to 0.1 kg, was used to measure body mass. These measures enabled the calculation of Body Mass Index (BMI) and BMI percentile. Waist circumference was measured at the narrowest point of the torso between the xiphoid process of the sternum and the iliac crest, to the nearest 0.1 cm. Skinfold measurements were completed by an anthropometrist, trained and qualified by the International Society for the Advancement of Kinathropometry (ISAK). Skinfold measurements were taken from the tricep, subscapular, supraspinale and front thigh using Harpenden Skinfold Callipers (Baty International, UK) and according to ISAK procedures. Furthermore, sitting height was measured and utilised to determine an estimation of maturity (by calculating years from peak height velocity)^26^. Immediately following the completion of anthropometric measures (~ 9.45 am), participants completed a Multi-Stage Fitness Test (MSFT); this enabled a measurement of cardiorespiratory fitness to be obtained for each participant^27^. Participants were also familiarised to The Daily Mile during the familiarisation trial.

### Study population and recruitment

Following ethical approval from the Nottingham Trent University School of Science and Technology Ethical Advisory Committee, primary schools within the East Midlands, UK were contacted via email and invited to participate in the study. The location of participating schools varied and included rural village, urban town and inner city. Six schools were implementing The Daily Mile at the time of the study and two schools had never implemented the initiative. The process of recruiting participants varied within each school, according to the preferences of the headteacher. However, headteachers were informed that a diverse sample of participants was required (i.e. varying PA, fitness, and academic level) and in most schools all children from years five and six (9–11 years old) were invited to participate.

In accordance with the guidelines for school-based research, headteacher consent was gained in addition to written informed consent from parents/guardians of participating children. A health screen questionnaire was also completed by parents/guardians to determine each child’s eligibility for participation, with any child with a health condition that may be affected by participating in the study (e.g. heart condition) excluded from participation in the study. Moreover, children provided their written assent to participate in the study. A total of 80 (40 female) primary school children aged 9–11 years participated in the study. However, due to technical issues with eight GPS units during data collection, data for 72 participants were included in the analysis. Participants from six schools were year five children (*n* = 52 [29 female], 10.1 ± 0.2 years) and participants from two schools were year six children (*n* = 20 [9 female], 11.3 ± 0.3 years).

### Data collection

#### Multi-stage fitness test

The MSFT is a valid and reliable measurement of cardiorespiratory fitness and accounts for individual training effects^27–29^. The MSFT involves participants completing progressive 20 m shuttle runs until volitional exhaustion, or until they are unable to keep time with the audio signal which dictates the required running speed. The running speed commences at 8.5 km^.^h^−1^ and increases by 0.5 km^.^h^−1^ for each 1 min stage completed^27^. Participants were paced by a member of the research team and verbal encouragement was provided by all members of the team throughout, in order to encourage maximal effort from the participants. Participants were assigned to a fitness quartile (quartile 1: lowest fit, 4: highest fit), based on distance covered (m) in the MSFT^30^. This split was performed according to sex, resulting in an equal number of boys and girls within each fitness quartile.

#### The Daily Mile

Participants were familiarised to The Daily Mile protocol during a familiarisation trial. During the main trial, participants completed The Daily Mile, which consisted of 20 min of outdoor activity, under the supervision of the researchers. The activity was self-paced; participants chose whether and when they walked, jogged, ran or sprinted. Participants completed The Daily Mile in groups of between 5–16 (mean: 12 ± 3) and were informed that they could exercise alone and/or with others. Moreover, participants wore normal school uniform with appropriate footwear. The exercise protocol was designed to mimic ‘The Daily Mile’, and other similar initiatives, currently being implemented in primary schools across the UK.

#### Heart rate and rating of perceived exertion

Heart rate (HR) was recorded during participation in The Daily Mile using a chest worn heart rate monitor (Firstbeat Technologies Ltd., Finland). Specifically, average and peak HR were calculated. Maximal HR was predicted using methods previously described^31^ and validated in children^32^. Average and peak HR were subsequently expressed as a percentage of maximum HR. Classification of the exercise intensity was then performed using American College of Sports Medicine (ACSM) guidelines^33^. Upon completion of The Daily Mile, participants were presented with the Children’s OMNI Scale in order to gain a valid and reliable rating of perceived exertion (RPE)^34–36^.

#### Global positioning systems

During participation in The Daily Mile, 15 Hz global positioning systems (GPS) (SPI HPU, GPSports, Canberra, Australia) were worn by each participant in a harness, which held the GPS unit in position between the shoulder blades. For all data, satellite coverage was 8 ± 1 satellites and horizontal dilution of precision was 0.48 ± 0.02. The variables of interest were: total distance covered, distance covered within age-specific speed zones and number of speed zone entries. These were calculated over the whole 20 min and within each 5 min split of The Daily Mile (0–5 min, 5–10 min, 10–15 min and 15–20 min), to examine whether activity patterns changed across the duration of The Daily Mile. Age group-specific speed zones were adapted from those published on youth soccer players^37^. The following speed zones were used: standing (≤ 0.1 m^.^s^−1^), walking (0.1–0.83 m^.^s^−1^), low-speed running (0.84–2.84 m^.^s^−1^), moderate-speed running (2.85–3.79 m^.^s^−1^), high-speed running (3.80–4.73 m^.^s^−1^), and sprinting (> 4.73 m^.^s^−1^). For the distance covered in each speed zone, standing and walking zones were combined. The number of speed zone entries was also used as a measure of the ‘nature’ of movement (i.e. continuous vs. intermittent). All GPS data were analysed using Teams AMS Software Version 1.2 (GPSports, Canberra, Australia). GPS are a valid and reliable tool for providing movement pattern data and this commercially available software has been used successfully in numerous studies on PA patterns and performance in youth^37–39^.

### Statistical analysis

Data were analysed in Statistical Package for the Social Sciences (SPSS) (Version 24; SPSS Inc., Chicago, IL., USA). Differences in HR and RPE between sexes were analysed using Mann–Whitney U test and Cohen’s d effect sizes were calculated. Moreover, differences between fitness quartiles in HR and RPE were analysed using Kruskall-Wallis H test. When analysing activity patterns (total distance covered, distance covered in each speed zone and number of speed zone entries) over time (i.e. for each 5 min split), one-way repeated measures analyses of variance (ANOVA) were conducted and partial eta squared effect sizes were calculated. Moreover, post-hoc Bonferroni pairwise comparison tests were used to examine the differences between each 5 min split and Cohen’s d effect sizes were calculated. To examine the effect of sex on activity patterns during The Daily Mile, independent samples t-tests were used and Cohen’s d effect sizes were calculated. To examine how activity patterns differed between the sexes in each 5 min split, two-way ANOVA (sex * split time; with repeated measures for split time) were conducted and partial eta squared effect sizes were calculated. To examine the effect of fitness quartile on activity patterns during The Daily Mile, one-way ANOVA were utilised and omega squared effect sizes were calculated. Bonferroni post-hoc tests were used to examine the differences between the fitness quartiles and Cohen’s d effect sizes were calculated. Moreover, to examine how activity patterns were affected by fitness in each 5 min split, two-way ANOVA (fitness * split time; with repeated measures for split time) were conducted and partial eta squared effect sizes were calculated. Statistical significance was accepted as *p* ≤ 0.05. Data are presented as mean ± standard deviation (SD).

## Results

### Participant characteristics

Participant characteristics are displayed in Table 1, for the sample overall and also split by boys and girls. On average, the children were 10.4 ± 0.7 years old and had a BMI percentile of 49.7 ± 28.1.

**Table 1:** Participant characteristics.

	Overall (n = 72)	Boys (n = 34)	Girls (n = 38)
Age (years)	10.4 ± 0.7	10.4 ± 0.7	10.4 ± 0.7
Height (cm)	143.9 ± 8.4	144.8 ± 7.7	142.9 ± 8.9
Body mass (kg)	36.3 ± 8.7	37.4 ± 9.5	35.2 ± 7.7
Body mass index (BMI) (kg^.^m^−2^)	17.3 ± 2.6	17.6 ± 2.8	17.0 ± 2.3
BMI percentile	49.7 ± 28.1	55.4 ± 28.6	44.5 ± 26.3
Waist circumference (cm)	61.9 ± 8.0	62.9 ± 8.4	60.6 ± 7.1
Sum of skinfolds (mm)	51.6 ± 24.1	51.8 ± 26.1	57.1 ± 23.4
Maturity offset (years)	− 2.0 ± 0.9	− 2.7 ± 0.6	− 1.4 ± 0.7

### Heart rate and rating of perceived exertion

During participation in The Daily Mile, average HR (main effect of sex, U = 103, *p* = 0.015, d = 0.67), peak HR (main effect of sex, U = 116, *p* = 0.038, d = 0.06) and the overall relative exercise intensity (main effect of sex, U = 147, *p* = 0.006, d = 0.61) were significantly higher in boys, compared to girls (Table 2). Moreover, there was a tendency for average HR to be highest in the highest fit (quartile 4) participants and lowest in the lowest fit (quartile 1) participants, however this effect did not reach statistical significance (*p* = 0.052; Table 2). Additionally, there was no difference in peak HR (*p* = 0.198) and overall relative exercise intensity was similar between fitness groups during The Daily Mile (*p* = 0.41; Table 2). Furthermore, no difference in RPE was observed between the sexes (*p* = 0.090) or fitness quartiles (*p* = 0.149; Table 2).

**Table 2:** Participants average and peak heart rate during The Daily Mile, and rating of perceived exertion.

	Average heart rate		Peak heart rate		Rating of perceived exertion
	beats·min^−1^	% HR_max_^a^	Beats·min^−1^	% HR_max_^a^	
Whole sample	163 ± 27	81 ± 13	193 ± 18	96 ± 9	5 ± 2
Sex					
Boys	172 ± 27	85 ± 14	194 ± 23	96 ± 11	5 ± 2
Girls	155 ± 24*	77 ± 12*	193 ± 11*	96 ± 6	6 ± 3
Physical fitness					
Quartile 1 (lowest fit)	145 ± 26	72 ± 13	187 ± 21	93 ± 11	6 ± 3
Quartile 2	166 ± 20	83 ± 10	197 ± 6	98 ± 3	5 ± 2
Quartile 3	166 ± 29	83 ± 15	189 ± 25	94 ± 12	4 ± 2
Quartile 4 (highest fit)	174 ± 30	87 ± 15	201 ± 4	100 ± 2	6 ± 2

^a^The percentage of age predicted maximum heart rate (HR_max_) during The Daily Mile. HR_max_ predicted based upon (HR_max_ = 208–0.7[age])^25^.

*Boys > girls, all *p* < 0.05.

### Distance covered

#### Whole sample

**Figure 1:**
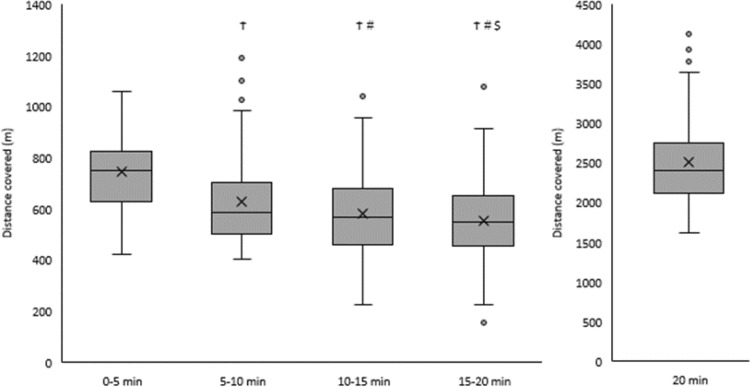
Distance covered (m) by participants during each 5 min split, and the total 20 min, of The Daily Mile. Boxplots represent the median (centre line), mean (cross), inter-quartile range (grey box), range (whiskers) and outliers (grey circles). Main effect of time, *p* < 0.001. ^Ϯ^Denotes significant difference from Split 1. ^#^Denotes a significant difference from Split 2. ^$^Denotes a significant difference from Split 3 (all *p* < 0.05).

Average distance covered by participants during The Daily Mile was 2511 ± 550 m (range 1616–4132 m). When considering the distance covered during each 5 min split, a significant main effect of time was observed (F_(2, 183)_ = 71.0, *p* < 0.001, η_p_^2^ = 0.473). Specifically, participants covered the greatest distance in the first 5 min of The Daily Mile (748 ± 141 m) and distance covered gradually decreased with each following 5 min split (5–10 min: 627 ± 160 m; 10–15 min: 582 ± 169 m; 15–20 min: 554 ± 162 m; Fig. 1).

#### Sex

**Figure 2:**
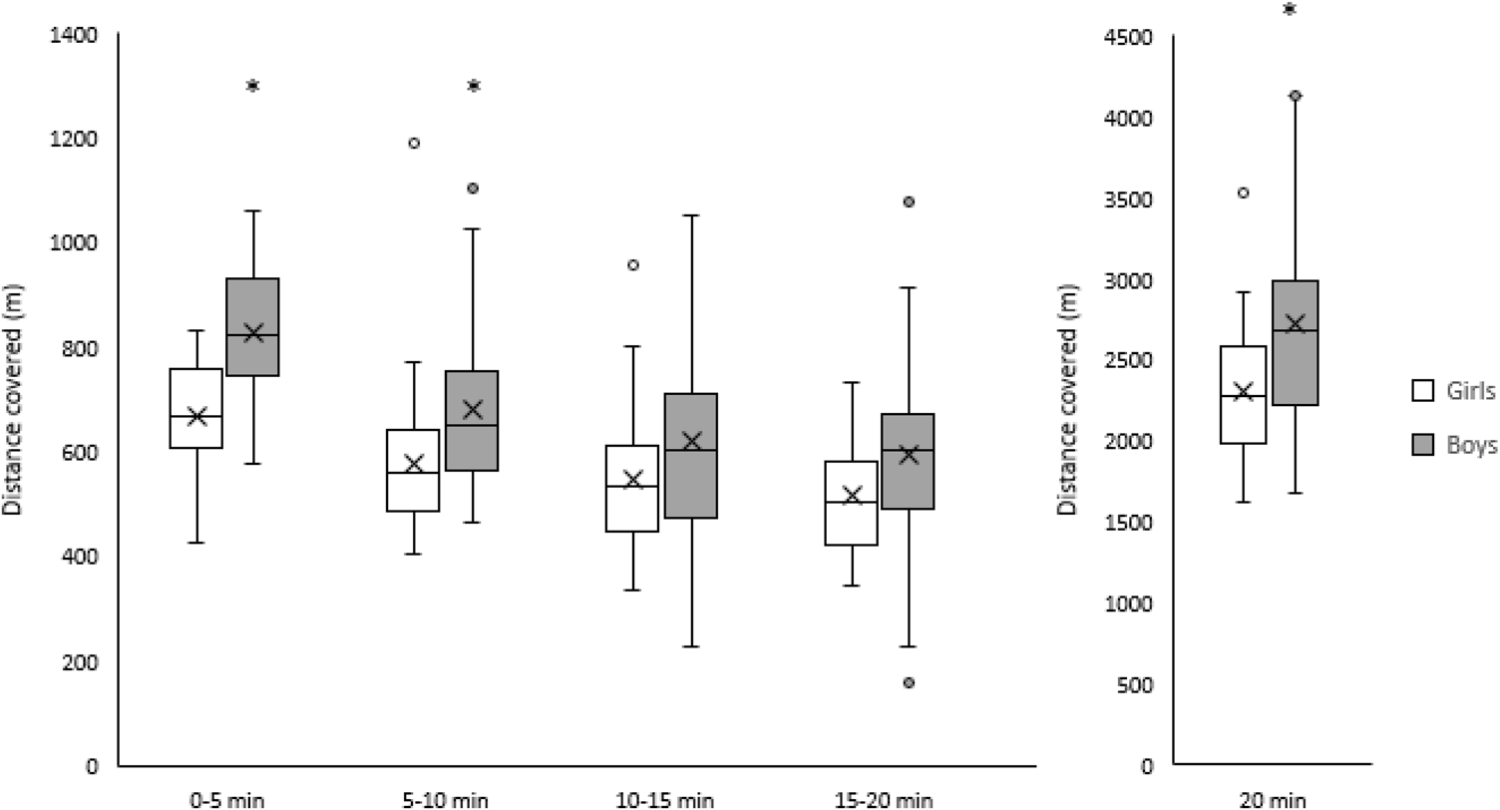
Distance covered (m) by boys and girls during each 5 min split, and the total 20 min of The Daily Mile. Main effect of sex, *p* = 0.001; sex by time interaction, *p* = 0.019. *Indicates distance covered by boys significantly greater than girls (*p* ≤ 0.05).

Total distance covered during the full 20 min of The Daily Mile was greater in boys compared to girls (Boys: 2717 ± 606 m, Girls: 2305 ± 398 m; main effect of sex, t_(67)_ = -3.6, *p* = 0.001, d = 0.80). Moreover, when considering the distance covered across each 5 min split, there was a significant sex by time interaction (F_(2, 193)_ = 3.7, *p* = 0.019, η_p_^2^ = 0.045). Specifically, the difference in distance covered between boys and girls was greatest in the first 5 min, and decreased across the remainder of The Daily Mile (Fig. 2).

#### Fitness quartile

**Figure 3:**
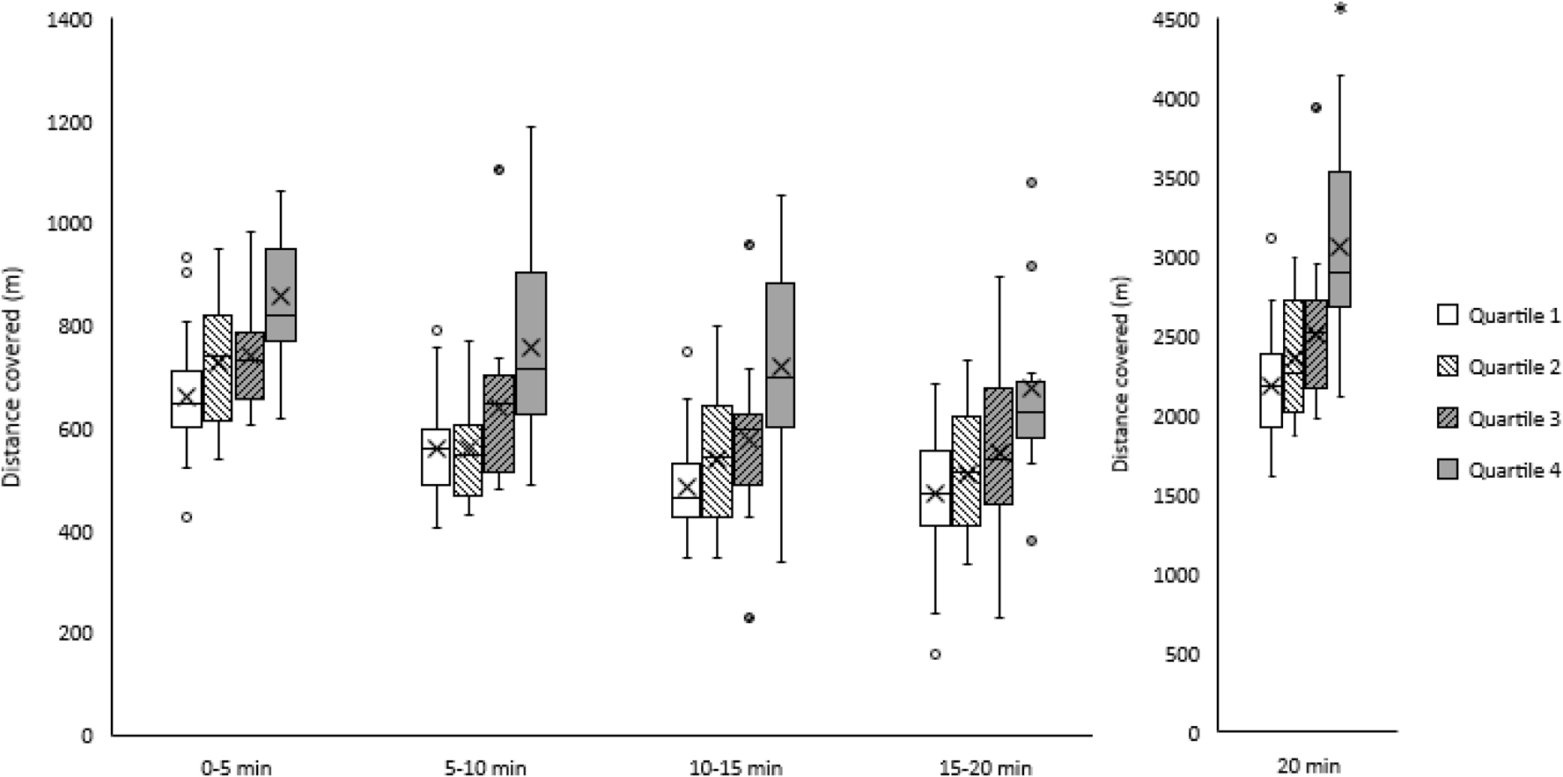
Distance covered (m) by participants in each fitness quartile (Quartile 1: lowest fit, Quartile 4: highest fit) during each 5 min split and the total 20 min of The Daily Mile. Main effect of fitness, *p* < 0.001. * Indicates distance covered by participants in quartile 4 greater than participants in quartiles 1, 2 and 3 (all *p* < 0.05).

Participant fitness had a significant effect on distance covered in The Daily Mile (main effect of fitness, F_(3, 68)_ = 10.4, *p* < 0.001, ω^2^ = 0.29). Specifically, participants in quartile 4 (highest fitness) covered a greater distance than quartile 1 (mean difference = 828 m, *p* < 0.001, d = 1.61), quartile 2 (mean difference = 673 m, *p* < 0.001, d = 0.25), and quartile 3 (mean difference = 494 m, *p* = 0.015, d = 0.90) participants (Fig. 3). However, there were no differences in distance covered between the other fitness quartiles (all *p* > 0.05), and there was no significant fitness by time interaction (*p* = 0.821).

### Distance covered in each speed zone

#### Whole sample

During the total 20 min of The Daily Mile, and within each 5 min split, participants covered the greatest distance in low speed running (speed zone 2) and moderate speed running (speed zone 3). The least distance was covered while walking (speed zone 1) and sprinting (speed zone 5). Moreover, distance covered in low speed running and sprinting remained relatively stable across each 5 min split of The Daily Mile (main effect of time, zone 2: *p* = 0.166; zone 5: *p* = 0.081). However, distance covered while walking varied significantly over time (F_(1, 115)_ = 10.0, *p* = 0.001, η_p_^2^ = 0.112). Specifically, distance covered while walking was lowest in the first 5 min (split 1 vs. 2: *p* = 0.002, d = 0.43; split 1 vs. 3, *p* = 0.002, d = 0.51; split 1 vs. 4: *p* < 0.001, d = 0.62) and highest in the last 5 min (split 2 vs. 4: *p* = 0.005, d = 0.44; split 3 vs. 4: *p* = 0.004, d = 0.34). Moreover, distance covered while running at a moderate speed changed significantly over time (F_(2, 173)_ = 77.7, *p* < 0.001, η_p_^2^ = 0.496), whereby distance covered at a moderate speed was greatest in the first 5 min (split 1 vs. 2: *p* < 0.001, d = 0.60; split 1 vs. 3: *p* < 0.001, d = 0.94; split 1 vs. 4: *p* < 0.001, d = 1.10) and decreased with each following 5 min (split 2 vs. 3: *p* = 0.001, d = 0.24; split 3 vs. 4: *p* = 0.003, d = 0.22). Furthermore, distance covered while running at a high speed varied significantly over time (F_(3, 205)_ = 7.7, *p* < 0.001, η_p_^2^ = 0.089). Specifically, distance covered in high speed running was greatest in the first 5 min (split 1 vs. 2: *p* < 0.001, d = 0.50; split 1 vs. 3: *p* < 0.001, d = 0.65; split 1 vs. 4: *p* = 0.014, d = 0.31), decreased and remained relatively stable for the middle 10 min, then increased significantly in the last 5 min of The Daily Mile (split 3 vs. 4: *p* = 0.047, d = 0.23) (Table 3).

**Table 3:** Distance covered (m) by participants in each speed zone during The Daily Mile.

		20 min	Split 1	Split 2	Split 3	Split 4
			0–5 min	5–10 min	10–15 min	15–20 min
**Walking (Zone 1) ≤ 0.83 m**^**.**^**s**^**−1**^						
All		37 ± 46	4 ± 6	7 ± 10^Ϯ^	10 ± 16^Ϯ^	15 ± 24^Ϯ #$^
Sex	Girls	36 ± 35	6 ± 6*	9 ± 11	8 ± 9	13 ± 15
	Boys	38 ± 55	3 ± 6	6 ± 9	13 ± 21	16 ± 31
Fitness	Q1	60 ± 45^a^	8 ± 7	11 ± 11	17 ± 12	24 ± 29
	Q2	37 ± 31	6 ± 8	8 ± 10	9 ± 10	14 ± 11
	Q3	38 ± 67	3 ± 4	5 ± 9	13 ± 28	17 ± 36
	Q4	19 ± 33	1 ± 2	5 ± 9	4 ± 8	8 ± 15
**Low speed running (Zone 2) 0.84–2.84 m**^**.**^**s**^**−1**^						
All		1370 ± 387	342 ± 128	356 ± 119	344 ± 105	328 ± 112
Sex	Girls	1469 ± 356*	375 ± 109	371 ± 111	370 ± 91*	352 ± 94
	Boys	1272 ± 396	309 ± 137	340 ± 126	318 ± 112	304 ± 124
Fitness	Q1	1366 ± 292	362 ± 110	370 ± 111	330 ± 63	304 ± 104
	Q2	1394 ± 275	347 ± 120	367 ± 105	352 ± 74	328 ± 79
	Q3	1539 ± 447^a^	402 ± 128	404 ± 118	374 ± 143	358 ± 125
	Q4	1127 ± 418	257 ± 121	276 ± 117	307 ± 122	286 ± 117
**Moderate speed running (Zone 3) 2.85–3.79 m**^**.**^**s**^**−1**^						
All		828 ± 663	326 ± 201	204 ± 194^Ϯ^	164 ± 181^Ϯ #^	134 ± 144^Ϯ#$^
Sex	Girls	560 ± 361	227 ± 126	134 ± 107	108 ± 98	91 ± 91
	Boys	1095 ± 784*	425 ± 215*	273 ± 235*	221 ± 225*	176 ± 174*
Fitness	Q1	562 ± 378^a^	237 ± 162	139 ± 102	95 ± 63	93 ± 88
	Q2	667 ± 411^a^	296 ± 168	152 ± 97	123 ± 115	96 ± 73
	Q3	624 ± 321^a^	254 ± 150	158 ± 99	112 ± 79	99 ± 75
	Q4	1461 ± 968	493 ± 221	362 ± 294	336 ± 286	270 ± 227
**High speed running (Zone 4) 3.80–4.73 m**^**.**^**s**^**−1**^						
All		195 ± 149	67 ± 59	41 ± 48^Ϯ^	37 ± 49^Ϯ^	49 ± 54^Ϯ$^
Sex	Girls	157 ± 126	53 ± 55	43 ± 53	30 ± 32	30 ± 34
	Boys	233 ± 161*	82 ± 60	39 ± 43	45 ± 61	67 ± 63*
Fitness	Q1	162 ± 148	9 ± 24	5 ± 16	3 ± 8	8 ± 17
	Q2	179 ± 143	7 ± 12	3 ± 5	23 ± 73	22 ± 69
	Q3	191 ± 167	11 ± 18	30 ± 98	38 ± 158	40 ± 134
	Q4	275 ± 137	10 ± 19	44 ± 115	33 ± 109	34 ± 89
**Sprinting (Zone 5) > 4.73 m**^**.**^**s**^**−1**^						
All		82 ± 252	8 ± 17	19 ± 72	26 ± 100	29 ± 89
Sex	Girls	84 ± 232	9 ± 20	18 ± 77	29 ± 94	28 ± 89
	Boys	80 ± 273	8 ± 15	20 ± 69	22 ± 106	29 ± 90*
Fitness	Q1	25 ± 48	9 ± 24	5 ± 16	3 ± 8	8 ± 17
	Q2	55 ± 107	7 ± 12	3 ± 5	23 ± 73	22 ± 69
	Q3	119 ± 402	11 ± 18	30 ± 98	38 ± 158	40 ± 134
	Q4	122 ± 137	10 ± 19	44 ± 155	33 ± 109	34 ± 89

*Q* quartile.

^Ϯ^Denotes significant difference (*p* ≤ 0.05) from Split 1.

^#^Denotes a significant difference from Split 2.

^$^Denotes a significant difference from Split 3.

*Indicates the sex that covered a significantly greater distance during the specified time frame.

^a^Represents a significant difference from quartile 4 participants.

#### Sex

Participant sex had a significant effect on distance covered in low, moderate and high speed running zones during The Daily Mile (main effect of sex, zone 2: t_(78)_ = 3.4, *p* = 0.022, d = 0.50; zone 3: t_(55)_ = − 3.9, *p* < 0.001, d = 0.9; zone 4: t_(78)_ = − 2.4, *p* = 0.020, d = 0.51). Specifically, girls covered a greater distance in low speed running compared to boys, whereas boys covered a greater distance in moderate and high speed running compared to girls (Table 3). Moreover, there was a significant sex by time interaction effect on distance covered in moderate speed (F_(2, 178)_ = 6.8, *p* = 0.001, η_p_^2^ = 0.081) and high speed (F_(3, 207)_ = 3.6, *p* = 0.018, η_p_^2^ = 0.044) running over time. Specifically, boys covered a greater distance in moderate speed running throughout each 5 min of The Daily Mile and in high speed running in the last 5 min, compared to girls (Table 3).

#### Fitness quartile

Participant fitness had a significant effect on distance covered in low and moderate speed running during The Daily Mile (main effect of fitness, zone 2: F_(3, 68)_ = 3.7, *p* = 0.015, ω^2^ = 0.14; zone 3: F_(3, 68)_ = 9.6, *p* < 0.001, ω^2^ = 0.27, Table 3). Specifically, participants in quartile 3 travelled significantly further in low speed running compared to quartile 4 participants (*p* = 0.009, d = 0.92). Quartile 4 participants, however, travelled significantly further in moderate speed running, compared to participants in all other fitness quartiles (quartile 1 vs. 4: *p* < 0.001, d = 1.21; quartile 2 vs. 4: *p* = 0.001, d = 1.10; quartile 3 vs. 4: *p* < 0.001, d = 1.23). Regarding the distance covered in each speed zone across the 5 min splits of The Daily Mile, no significant fitness by time interaction effects were observed (zone 1: *p* = 0.623; zone 2: *p* = 0.162; zone 3: *p* = 0.602; zone 4: *p* = 0.377; zone 5: *p* = 0.692).

### Speed zone entries

#### Whole sample

During participation in The Daily Mile, the mean number of speed zone entries for each participant was 646 ± 175. There was, however a large variance between participants in the number of speed zone entries (range 156–1365) (Table 4). Moreover, the number of speed zone entries made by participants during The Daily Mile differed over time (F_(3, 206)_ = 38.2, *p* < 0.001, η_p_^2^ = 0.326). Specifically, participants completed the greatest number of entries in the first 5 min of The Daily Mile when compared to all other 5 min splits (split 1 vs. 2: *p* < 0.001, d = 0.94; split 1 vs. 3: *p* < 0.001, d = 0.94; split 1 vs. 4: *p* < 0.001, d = 0.83, Table 4).

**Table 4:** Mean ± SD (range) speed zone entries made by participants during The Daily Mile.

	20 min	Split 1	Split 2	Split 3	Split 4
		0–5 min	5–10 min	10–15 min	15–20 min
All	646 ± 275 (156–1365)	217 ± 79 (63–426)	148 ± 75^Ϯ^ (13–406)	139 ± 87^Ϯ^ (5–485)	146 ± 90^Ϯ^ (7–445)
**Sex**					
Girls	523 ± 218 (156–1227)	179 ± 65 (75–322)	121 ± 65 (13–274)	108 ± 58 (5–321)	118 ± 76 (7–365)
Boys	770 ± 274* (362–1365)	255 ± 74 (63–426)	175 ± 75 (73–406)	170 ± 99 (38–485)	173 ± 94 (73–445)
**Fitness**					
Q1	632 ± 282 (258–1227)	193 ± 78 (84–380)	138 ± 68 (47–274)	153 ± 72 (29–321)	151 ± 100 (7–365)
Q2	594 ± 196 (253–976)	219 ± 67 (75–322)	140 ± 67 (19–271)	115 ± 68 (34–286)	124 ± 60 (25–241)
Q3	589 ± 277 (156–1295)	197 ± 82 (90–340)	131 ± 72 (13–261)	127 ± 68 (5–269)	137 ± 93 (25–428)
Q4	766 ± 316 (362–1365)	243 ± 78 (63–423)	183 ± 72 (82–285)	154 ± 106 (42–364)	190 ± 106 (42–445)

*Q* Quartile.

^Ϯ^Denotes a significant difference (*p* ≤ 0.05) from Split 1.

*Indicates number of speed zone entries by boys significantly greater than girls.

#### Sex

Boys completed significantly more speed zone entries compared to girls during the total 20 min of The Daily Mile (main effect of sex, t_(78)_ = − 4.8, *p* < 0.001, d = 1.04). There were, however, large ranges in the mean number of zone entries within each sex (Girls: 156–1227; Boys: 362–1365, Table 4). Moreover, there was no difference in the number of entries made between sexes within any of the 5 min splits (sex * time interaction, *p* = 0.343).

#### Fitness quartile

The highest fit (quartile 4) and lowest fit (quartile 1) participants presented the greatest mean number of zone entries during the 20 min of The Daily Mile (Table 4). However, there were no significant differences in the number of entries made during the total 20 min (main effect of fitness, *p* = 0.198) or each 5 min split (fitness * time interaction, *p* = 0.393) between fitness quartiles.

## Discussion

This is the first study to quantify the activity patterns (including distance covered in different speed zones and the number of speed zone entries) alongside the physiological responses of children participating in The Daily Mile. The total distance covered was 2511 m (~ 1.5 miles), mostly while running at low and moderate speeds. Average HR was 163 ± 27 beats·min^−1^, peak HR was 193 ± 18 beats·min^−1^, the relative exercise intensity was 81 ± 13% of age-predicted HR_max_ and average RPE was 5 ± 2 (which equates to ‘tired’) upon completion of The Daily Mile. Participants covered the greatest distance, and were also most intermittent, during the first 5 min of The Daily Mile, compared to the later stages. Moreover, boys and the highest fit children ran further and faster compared to girls and less fit children. Boys’ average HR, peak HR and their relative exercise intensity was higher than in girls, and boys’ activity was also more intermittent.

Although the children covered an average distance of 2511 m, equating to approximately 1.5 miles, there was a large range in the distances covered by participants, from ~ 1 mile to 2.5 miles, between boys and girls and between high fit and low fit participants. It has been previously suggested that the ‘dose’ of PA is a key determinant of the subsequent effects on participant health and cognition^8,40,41^ and thus it is possible the health and cognition benefits would vary between participants in the present study. However, whilst the relative exercise intensity was higher in boys compared to girls, the relative exercise intensity (average 81 ± 13%) did not differ between low and high fit children and all participants ran at least one mile; suggesting a potentially similar and positive health benefit for children of all fitness levels participating in The Daily Mile.

In the present study, the mean number of speed zone entries for each participant was 646 (a high number of entries demonstrating that participants frequently changed pace), so although there was a wide range of speed zone entries between participants from 156 to 1365 entries, all children were to some extent intermittent in their activity patterns during The Daily Mile. These findings support previous research suggesting that children typically choose to engage in sporadic, high intensity intermittent activity during discretionary exercise^42,43^. This intermittent choice of activity may be based on enjoyment, as it has been previously shown in young people aged 12–15 that enjoyment ratings are higher for intermittent than for continuous cycling exercise, and that enjoyment remains high even when bouts of exercise are at near maximal intensity^44,45^. Giving young people the choice to stop and start and to speed up and slow down during The Daily Mile may also be important for physiological health and cognitive function benefits, as glycaemic control and executive function were better in adolescents following intermittent in comparison with continuous exercise^46,47^. Therefore, the findings of the present study support the use of The Daily Mile as an intermittent form of activity in young people.

With regards to how activity patterns changed over time, during the first 5 min of The Daily Mile, children covered a higher total distance and a higher distance in the moderate- and high-speed running zones in comparison with the remaining 5 min splits. Furthermore, their activity was also the most intermittent during the first 5 min, as demonstrated by a higher number of speed zone entries. This information is valuable to school staff because it demonstrates that even if only 5 min can be spared on any given day (e.g. due to time constraints or weather concerns), it is worth using this time to implement The Daily Mile, as exercise during this time is of a high ‘dose’, and thus could still be beneficial for the young people. The total distance covered and distance covered while running at a moderate speed gradually decreased with each following 5 min, while distance covered while walking gradually increased. These changes are likely due to participants experiencing rising levels of fatigue as The Daily Mile progressed^48^. Moreover, while distance covered in high speed running remained stable during the middle 10 min of The Daily Mile, distance covered in this zone increased slightly in the last 5 min. Additionally, speed zone entries, and thus intermittent activity, decreased over the middle 10 min of The Daily Mile but increased slightly in the last 5 min. These findings suggest that children may invest additional effort when they know that they are approaching the end of a bout of PA; thus providing young people with information that there is 5 min remaining during The Daily Mile may increase the distance covered towards the end of the activity.

When examining the effect of participant cardiorespiratory fitness, as measured by the multi-stage fitness test in the present study, on activity patterns in The Daily Mile, participants in quartile 4 (highest fitness) covered between ~ 800 m and ~ 500 m more than participants in quartiles 1, 2 and 3. The intermittent/continuous nature of activity, however, was not affected by cardiorespiratory fitness. These findings highlight the importance of considering each participant’s physical fitness when evaluating the effectiveness of PA interventions. Importantly, although there was a tendency for the highest fit children to have the highest average HR, and for the lowest fit children to have the lowest average HR, there were no statistically significant differences in HR (average and peak), or RPE, between fitness quartiles during participation in The Daily Mile. Thus, while the absolute dose of activity may be different between children of different fitness levels (as the highest fit children tend to run further and faster), the relative dose of the activity is similar. This is an important novel finding of the present study given that the relative, compared to absolute, dose of PA is more likely to determine the physiological responses^49^. The Daily Mile initiative is, therefore, advantageous as children of all fitness levels are able to participate, receive a similar ‘relative’ dose of PA and are thus likely to gain similar benefits for health and cognitive function.

Participant sex was a significant moderator of activity patterns during The Daily Mile. Boys covered a greater distance compared to girls overall, and within each 5 min split of The Daily Mile. Moreover, whilst girls covered a greater distance running at a low-speed, boys covered a greater distance while running at faster speeds. Overall, this resulted in boys running further than girls, whose activity was slower paced. This greater distance covered by the boys would be expected due to their higher V̇O_2_ peak (e.g. 16% higher in 8–11 year old boys compared to girls)^50^ and their better performance in the multi-stage fitness test in the present study which may reflect a combination of both a higher V̇O_2_ peak and a better training status. Moreover, the boys’ average HR (172 ± 27 beats⋅min^−1^) was higher than girls (155 ± 24 beats⋅min^−1^) during The Daily Mile, reflecting a higher relative exercise intensity for the boys, which supports the suggestion that the boys were better trained. In addition, the boys could have chosen to undertake more exercise and a different type of exercise. Previous literature demonstrates that boys tend to view unstructured exercise time (e.g. recess) as an opportunity to engage in competitive behaviours, whereas girls tend to engage in social behaviours^51^. A recent ethnographic study on The Daily Mile reported that girls interacted more during participation, linking arms and chatting, and this was suggested to reduce MVPA^52^. Therefore, sex-specific social behaviours, in addition to differences in V̇O_2_ peak and training status, may also be responsible for the differences in activity patterns observed between the sexes in the present study. Furthermore, evidence suggests that boys experience greater improvements in cardiorespiratory fitness from chronic participation in school-based PA interventions^53^. The greater relative exercise intensity observed in boys compared to girls during The Daily Mile may therefore result in boys gaining greater benefits to health and cognitive function from participation over time.

Previous research has highlighted that more in-school PA opportunities are necessary, as not enough children are meeting the daily 30 min MVPA target^13^. Notwithstanding the differences in activity patterns between boys and girls and children of different fitness levels, the findings of the present study demonstrate that The Daily Mile is an effective intervention which allows all young people to accrue PA during the school day. Specifically, in the 20 min Daily Mile in the present study, all young people covered at least one mile. However, while the findings suggest that The Daily Mile contributes towards this target, the contribution is more significant for boys. Moreover, current evidence on the chronic effects of The Daily Mile on children’s cardiorespiratory fitness is contradictory. While some studies found improvements to cardiorespiratory fitness at 12 weeks^54–56^, others reported no effects to cardiorespiratory fitness following six^57^ or 12 months of participation^58^. Future research could thus consider whether making adaptations to The Daily Mile and/or implementing a different activity may be more successful in contributing to these targets and improving cardiorespiratory fitness in both sexes. For example, previous observational research on The Daily Mile has shown that children spend the greatest time performing MVPA during The Daily Mile when it is being promoted by teachers^12^. Therefore, school staff implementing The Daily Mile could focus on encouraging girls to engage in more physical activity during participation to ameliorate the differences in activity patterns between boys and girls demonstrated in the present study. Alternatively, perhaps an activity with slightly more structure may be more effective in eliciting a greater activity engagement from girls. It must, however, be remembered that the simple and social nature of The Daily Mile are factors considered core to its popularity and success as a school-based intervention^12,59^.

While this paper has several strengths, it is not without limitations. For example, it is possible that the recruitment process resulted in selection bias, with less PA-focused schools and less fit children opting out of participation. However, the schools which participated in the study varied in size (105–660 pupils) and location (rural village—inner city), and whether they had previously implemented any active mile initiatives. Additionally, participating children displayed a wide range of cardiorespiratory fitness, body mass and adiposity; thus representing a diverse sample. Nevertheless, our data should be interpreted with the potential selection bias in mind. Moreover, the findings of the present study are specific to a 20 min Daily Mile. Whilst we feel that the activity patterns of children will be similar with other durations (e.g. 15 min), the exact effects warrant further investigation. Furthermore, group sizes during participation in The Daily Mile varied (5–16 children) due to the logistical challenges of conducting the testing within each school and it is possible that the activity patterns of children may differ when participating in larger groups such as a whole class. However, the present study provides novel quantitative insight into the activity patterns of young people participating in The Daily Mile.

## Conclusions

This study is the first to report activity patterns during The Daily Mile. On average, participants covered ~ 1.5 miles and exercised at a moderate-to-vigorous intensity. Furthermore, the findings of the present study demonstrate that boys run further and faster than girls, and that boy’s activity is more intermittent, during The Daily Mile. Moreover, this study demonstrates that whilst high fit children run further and faster than lower fit children during The Daily Mile, the relative exercise intensity is similar between fitness groups; this suggests that The Daily Mile facilitates a similar relative ‘dose’ of PA for children of all fitness levels. Furthermore, by quantifying the dose and nature of PA during The Daily Mile, this study has enabled a greater understanding of how acute and chronic participation may impact children’s health and cognition. Future research should seek to explore how children’s activity patterns change over time with regular participation in The Daily Mile and should examine the potential moderating role of sex and cardiorespiratory fitness in these relationships.

## Data Availability

The datasets generated and/or analysed during the current study are available from the corresponding author on reasonable request.
